# An Inflammatory Response Related Gene Signature Associated with Survival Outcome and Gemcitabine Response in Patients with Pancreatic Ductal Adenocarcinoma

**DOI:** 10.3389/fphar.2021.778294

**Published:** 2021-12-23

**Authors:** Zhijun Xiao, Jinyin Li, Qian Yu, Ting Zhou, Jingjing Duan, Zhen Yang, Cuicui Liu, Feng Xu

**Affiliations:** ^1^ Department of Pharmacy, Shanghai University of Medicine and Health Sciences Affiliated Sixth People’s Hospital South Campus, Shanghai, China; ^2^ Department of Pharmacy, Xuhui Central Hospital of Shanghai, Shanghai, China; ^3^ Division of Interventional Radiology, University of Chicago, Chicago, IL, United States; ^4^ Department of Central Laboratory, Shanghai University of Medicine and Health Sciences Affiliated Sixth People’s Hospital South Campus, Shanghai, China; ^5^ Department of Clinical Laboratory, Shanghai University of Medicine and Health Sciences Affiliated Sixth People’s Hospital South Campus, Shanghai, China; ^6^ Department of Pharmacy, Fengxian Hospital, Southern Medical University, Shanghai, China

**Keywords:** pancreatic ductal adenocarcinoma, inflammatory response, gene signature, tumor microenvironment, gemcitabine response, gene mutation

## Abstract

**Background:** Pancreatic ductal adenocarcinoma (PDAC) is one of the most aggressive tumors with an extremely low 5-year survival rate. Accumulating evidence has unveiled that inflammatory response promotes tumor progression, enhances angiogenesis, and causes local immunosuppression. Herein, we aim to develop an inflammatory related prognostic signature, and found it could be used to predict gemcitabine response in PDAC.

**Methods:** PDAC cohorts with mRNA expression profiles and clinical information were systematically collected from the four public databases. An inflammatory response related genes (IRRGs) prognostic signature was constructed by LASSO regression analysis. Kaplan–Meier survival analysis, receiver operating characteristic analysis, principal component analysis, and univariate and multivariate Cox analyses were carried out to evaluate effectiveness, and reliability of the signature. The correlation between gemcitabine response and risk score was evaluated in the TCGA-PAAD cohort. The GDSC database, pRRophetic algorithm, and connectivity map analysis were used to predict gemcitabine sensitivity and identify potential drugs for the treatment of PDAC. Finally, we analyzed differences in frequencies of gene mutations, infiltration of immune cells, as well as biological functions between different subgroups divided by the prognostic signature.

**Results:** We established a seven IRRGs (ADM, DCBLD2, EREG, ITGA5, MIF, TREM1, and BTG2) signature which divided the PDAC patients into low- and high-risk groups. Prognostic value of the signature was validated in 11 PDAC cohorts consisting of 1337 PDAC patients from 6 countries. A nomogram that integrated the IRRGs signature and clinicopathologic factors of PDAC patients was constructed. The risk score showed positive correlation with gemcitabine resistance. Two drugs (BMS-536924 and dasatinib) might have potential therapeutic implications in high-risk PDAC patients. We found that the high-risk group had higher frequencies of KRAS, TP53, and CDKN2A mutations, increased infiltration of macrophages M0, neutrophils, and macrophages M2 cells, as well as upregulated hypoxia and glycolysis pathways, while the low-risk group had increased infiltration of CD8^+^ T, naïve B, and plasma and macrophages M1 cells.

**Conclusion:** We constructed and validated an IRRGs signature that could be used to predict the prognosis and gemcitabine response of patients with PDAC, as well as two drugs (BMS-536924 and dasatinib) may contribute to PDAC treatment.

## Introduction

Pancreatic cancer is a devastating digestive system malignant tumor characterized by limited treatment success and dismal prognosis. It accounts for 466,003 deaths in 185 countries in 2020 and is the seventh leading cause of death from cancer in both genders (Sung et al., 2021). It has been estimated that pancreatic cancer will surpass breast cancer as the third leading cause of cancer death after lung and colorectal cancers by 2025 in the European Union (Ferlay et al., 2016) and become the second most common cause of cancer-related death in the United States to the year 2040 (Rahib et al., 2021). Pancreatic ductal adenocarcinoma (PDAC), a type of pancreatic exocrine tumor, accounts for more than 90% of all pancreatic cancer (Grossberg et al., 2020). Despite progress in our understanding of pathogenesis of PDAC, the actual 5-year survival rate of this disease is below 5%, which has not improved over the past decades (Bengtsson et al., 2020). In the clinical setting, the prognostic prediction of PDAC mainly depends on tumor stage classified by the TNM staging system. However, this system is limited by the lack of consideration of the tumor biological heterogeneity at the molecular level. As such, it is imperative to explore novel signatures to predict prognostic and offer individualized treatment modalities for PDAC patients.

Inflammation has been regarded as a significant protective response that plays a pivotal role during regeneration of tissue injuries (Karin and Clevers, 2016). However, sustained inflammatory stimuli could result in chronic inflammation and impaired tissue regeneration (Hibino et al., 2021). This process is also associated with the development of a spectrum of diseases, such as cardiovascular disease, neurodegenerative disorders, and various types of cancer (Furman et al., 2019). Researchers noted the link between cancer and inflammation as early as 1863 (Balkwill and Mantovani, 2001). Tumor-promoting inflammation had been one of the newly added hallmarks of cancer (Hanahan and Weinberg, 2011). That inflammation provides a tumor-supportive microenvironment to stimulate tumor formation, growth, progression, and metastasis has been demonstrated in multiple studies (Wang and DuBois, 2015). Previous evidence also revealed that inflammatory responses play important roles at different stages of cancer development (Grivennikov et al., 2010). Furthermore, clinical and laboratory research suggested that use of anti-inflammatory agents is a promising approach for cancer prevention and treatment (Zappavigna et al., 2020). Blockade of inflammatory cytokine IL-6 could modulate immunological features of PDAC and enhance the efficacy of anti-PD-L1 therapy (Mace et al., 2018). Additionally, biomarkers of inflammatory response have been demonstrated with prognostic value in multiple cancers. C-reactive protein has been proved as a prognostic indicator for survival of patients with pancreatic cancer, as well as other types of cancer (Szkandera et al., 2014). High neutrophil-lymphocyte ratio and high platelet-lymphocyte ratio are important predictors of poor survival in patients with resectable PDAC (Ye and Bai, 2018). Several recent studies have demonstrated that inflammatory response related genes (IRRGs) could be used to predict the prognosis of hepatocellular carcinoma, transitional bladder cancer, and low-grade glioma (Han et al., 2021; Lin et al., 2021; Xie et al., 2021). However, the relationship between IRRGs and the prognosis of PDAC patients still needs to be clarified.

**FIGURE 1:**
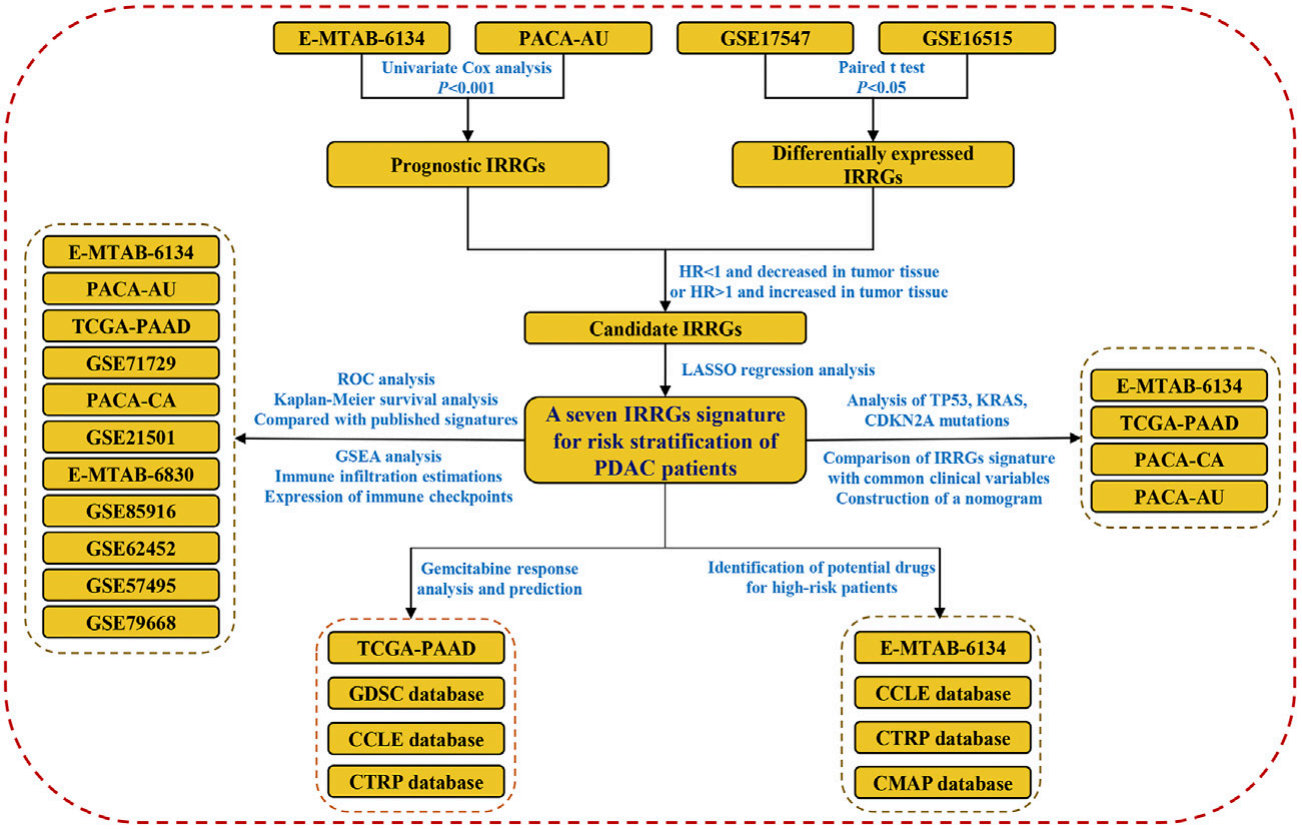
Flow chart of this study.

Herein, we systematically collected PDAC cohorts with gene expression data and clinical information from TCGA, ICGC, ArrayExpress, and GEO databases. Subsequently, an IRRGs signature for risk stratification of PDAC patients was constructed and validated. A nomogram which had high predictive value was constructed. We identified potential drugs for the treatment of high-risk patients since they were more likely resistant to gemcitabine. Finally, we compared the difference of TP53, KRAS, and CDNA2A mutations, infiltration of immune cells, as well as biological function between high- and low-risk groups. The flow chart of this work is shown in Figure 1.

## Materials and Methods

### Collection of PDAC Cohorts with Clinical and Gene Expression Data

Clinical and gene expression data of patients who had a pathological diagnosis of PDAC were systematically collected from TCGA, ICGC, GEO, and ArrayExpress databases. Patients who survived less than 60 days were removed from the cohorts, as these deaths might be a sequela of post-surgical complication, cardiovascular disease, and mortality other than cancer. As a result, a total of 11 PDAC cohorts contained 1337 PDAC patients were obtained. Detailed clinical information of the 11 cohorts are presented in Supplementary Table S1.

### Identification of Overall Survival-Related IRRGs

The IRRGs were collected from the “Hallmark inflammatory response” and “GOBP inflammatory response” gene sets in the Molecular Signatures database (MSigDB) (http://www.gsea-msigdb.org/) (Subramanian et al., 2005; Liberzon et al., 2015). To identify IRRGs associated with patients’ overall survival (OS) in E-TMAB-6134 and PACA-AU cohorts, the univariate Cox regression analysis was carried out with a *p*-value threshold of 0.001 by using the R package “survival.” The R package “VennDiagram” was then used to screen intersecting genes, which were selected as OS-related IRRGs. In order to determine which OS-related IRRGs may play a role in PDAC development, we then compared their expression levels between tumor and paired adjacent tissue in two GEO cohorts (GSE15471 and GSE16515). Taken together, genes with HR <1 and decreased expression in tumor or genes with HR >1 and increased expression in tumor were used as candidates for the signature construction.

### Establishment and Validation of a Prognostic IRRGs Signature

The LASSO regression analysis (Tibshirani, 1997), which was designed for variable selection and shrinkage, was conducted by using the R package “glmnet” to construct an IRRGs prognostic signature. In LASSO, variables with a regression coefficient equal to zero after the shrinkage process are excluded from the model. A tuning parameter lambda (*λ*) controls the amount of shrinkage, with increased shrinkage for higher *λ* values. An optimal *λ* was chosen when the partial likelihood deviance reached its lowest, which was based on the 10-fold cross-validation. The risk score of the signature for each patient was calculated as follows:

$$Risk\;score=\overset{n}{\underset{i=1}{\sum }}\left(Exp_{i}*\beta_{i}\right)$$

where *n* represents the number of prognostic genes, *Exp*_*i*_ represents the expression value of gene *i,* and *β*_*i*_ represents the regression coefficient of gene *i*.

We selected E-MTAB-6134 cohort as a training set because it contains the largest number of patients among all cohorts. Other 10 cohorts were used as validation sets to validate reliability of the signature. In each cohort, patients were divided into the high- or low-risk group according to the median value of the risk score. To compare the survival difference between high- and low-risk groups, Kaplan–Meier survival and receiver operating characteristic (ROC) analyses were conducted by using the R package “survival” and “timeROC.” We also calculated the time-varying concordance index (C-index) of our signature and compared it with six previous published signatures which were based on glycolysis (Song et al., 2021), ferroptosis (Feng et al., 2021a), hypoxia (Ding et al., 2021), m6A (Meng et al., 2020), autophagy (Yu J. et al., 2021), and immune (Mao et al., 2021) related genes. The information of these six signatures are listed in Supplementary Table S2. Furthermore, to verify the IRRGs signature was independent of other clinical variables (tumor grade, tumor stage, and resection margin) for the OS prediction, univariate and multivariate Cox regression analyses were performed in cohorts with clinical variables documented.

### Correlation Between Gemcitabine Response and Risk Score

Gemcitabine response of patients in TCGA-PAAD cohort was obtained by using R package TCGAbiolinks. Patients’ risk scores were compared among four types of gemcitabine response, including complete response (CR), partial response (PR), stable disease (SD), and progressive disease (PD). Furthermore, risk scores of 808 cancer cell lines in Genomics of Drug Sensitivity in Cancer (GDSC) (Yang et al., 2013) were calculated by the IRRGs signature. The correlation between IC50 value of gemcitabine and risk score of cancer cell lines was explored.

### Identification of Potential Drugs

Expression data of human cancer cell lines (CCLs) were downloaded from the Broad Institute Cancer Cell Line Encyclopedia (CCLE) project (https://portals.broadinstitute.org/ccle/) (Ghandi et al., 2019). Drug sensitivity data of CCLs were extracted from the Cancer Therapeutics Response Portal (CTRP v.2.0, https://portals.broadinstitute.org/ctrp) (Rees et al., 2016). The area under the dose-response curve (AUC) was used as a measure of drug sensitivity in CTRP, and higher AUC values indicate decreased sensitivity to drugs. Based on drug sensitivity and gene expression data from CTRP, drug response of patients was predicted according to their gene expression levels by using the R package pRRophetic (Geeleher et al., 2014). The AUC value of each drug in each patient was estimated. Additionally, the connectivity map (CMap) analysis (Subramanian et al., 2017) was also used to predicted potential small molecule drugs for the treatment of high-risk PDAC patients.

### Comparison of KRAS, TP53, and CDKN2A Mutations in High- and Low-Risk Patients

Somatic mutations of the KRAS, TP53, and CDKN2A are the common events in PDAC patients, and KRAS and TP53 mutations have been tightly linked to tumor-promoting inflammation (Yachida et al., 2012; Uehara and Tanaka, 2018; Hamarsheh et al., 2020). Thus, we compared the frequencies of KRAS, TP53, and CDKN2A mutations between high- and low-risk PDAC patients. The Mutation Annotation Format (MAF) files of TCGA-PAAD cohort were downloaded from the GDC database (https://portal.gdc.cancer.gov/). The simple somatic mutation format files of PACA-AU and PACA-CA cohorts were obtained from ICGC database (https://dcc.icgc.org/) and converted to MAF files by using R package “matfools” (Mayakonda et al., 2018). Likewise, all the MAF files were analyzed by using “matfools” package. Additionally, we extracted the KRAS, TP53, and CDKN2A mutation status of patients in E-MTAB-6134 cohort, which were documented in a clinical information file.

### Immune Infiltration Estimations and Gene Set Enrichment Analysis

Next, to gain insight into the difference in tumor immune microenvironment between high- and low-risk groups, CIBERSORT algorithm (https://cibersortx.stanford.edu/) (Chen et al., 2018) was used to estimate the proportion of 22 immune cells in each sample based on their gene expression profiles. Then, we quantitatively compared the infiltration of 22 immune cells between high- and low-risk groups. The estimate algorithm was also used to calculate stromal and immune scores of each sample by using R package estimate. Gene Set Enrichment Analysis (GSEA) was conducted to explore the biological difference between high- and low-risk groups. The analysis was performed by using GSEA software, and false discovery rate (FDR) < 0.25 was regarded as statistically significant according to the GSEA user guide (Subramanian et al., 2005).

## Results

### Identification of OS-Related IRRGs

**FIGURE 2:**
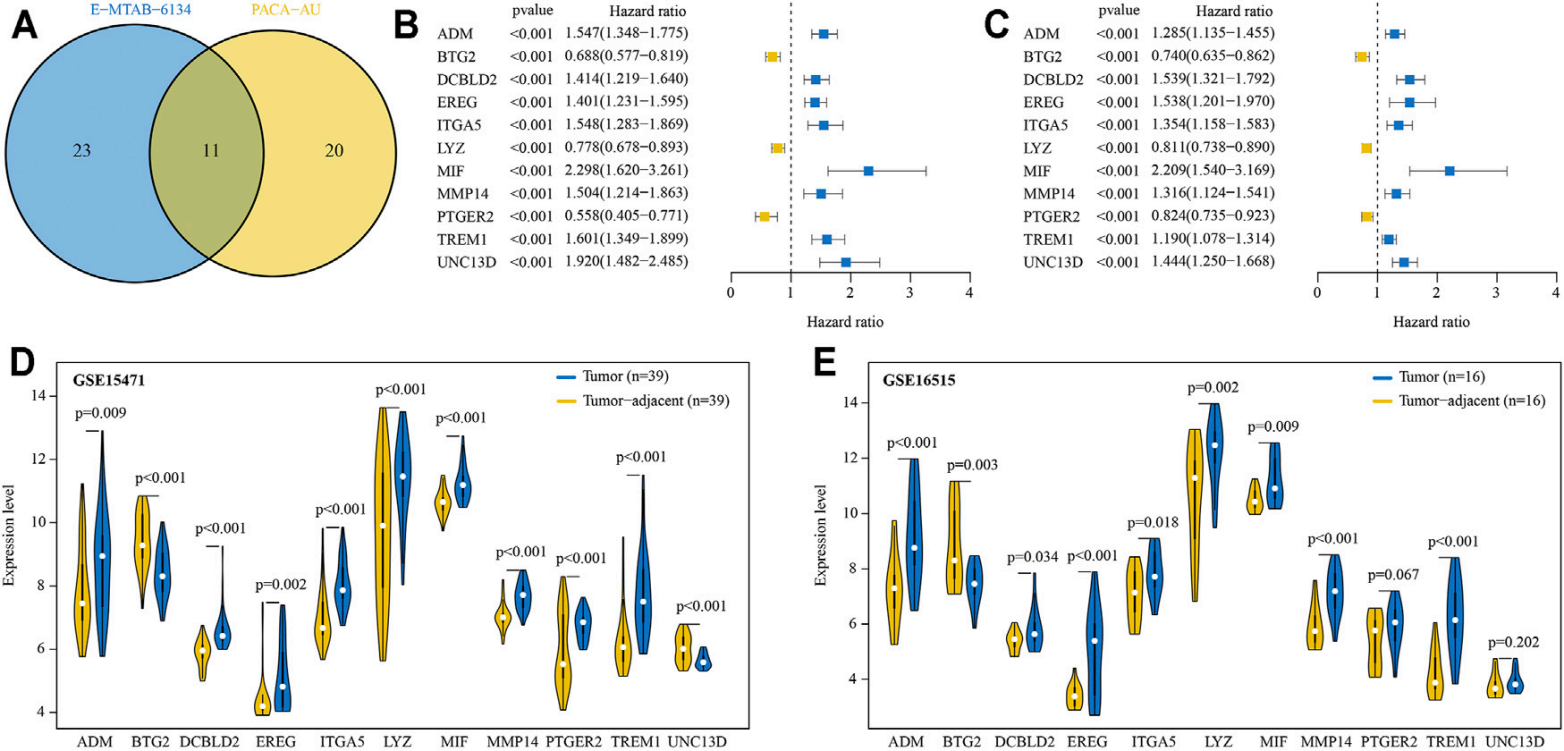
Prognostic IRRGs for developing a risk signature were identified. **(A)** A total of 11 IRRGs were screened out by Venn diagram. **(B, C)** The hazard ratio and 95% confidence intervals of 11 IRRGs in E-MTAB-6134 **(B)** and PACA-AU **(C)** cohorts. **(D, E)** Compared expression levels of 11 IRRGs between tumor and paired adjacent normal tissue in GSE15417 **(D)** and GSE16515 **(E)** cohorts.

There were 717 and 200 protein coding genes contained in the “GOBP inflammatory response” and “Hallmark inflammatory response” gene sets, respectively (Supplementary Tables S3 and S4). After removing the overlapped genes, a total of 841 IRRGs were obtained for further analysis. Univariate Cox regression analysis showed that there were 34 and 31 genes significantly (*p* < 0.001) related to OS of PDAC patients in E-MATB-6134 and PACA-AU cohorts, respectively (Supplementary Tables S5 and S6). A total of 11 intersecting genes were identified by using Venn diagram (Figure 2A). Subsequently, expression levels of these 11 genes were compared between tumor and tumor-adjacent tissues in two GEO cohorts. Taken together, eight genes were selected for LASSO analysis. Of these, ADM, DCBLD2, EREG, ITGA5, MIF, MMP14, and TREM1 were associated with poor prognosis and significantly increased in tumor, while BTG2 was related to favorable prognosis and decreased in tumor (Figures 2B–E).

### Construction of IRRGs Prognostic Signature

A prognostic signature containing seven IRRGs was constructed based on the optimal value of penalty lambda (Supplementary Figures S1A,B). The risk score of each patient was calculated as follows: risk score = expression level of ITGA5*0.059 + expression level of TREM1*0.109 + expression level of EREG*0.132 + expression level of MIF*0.216 + expression level of ADM*0.204 + expression level of DCBLD2*0.129-expression level of BTG2*0.098 (Supplementary Figure S1C).

**FIGURE 3:**
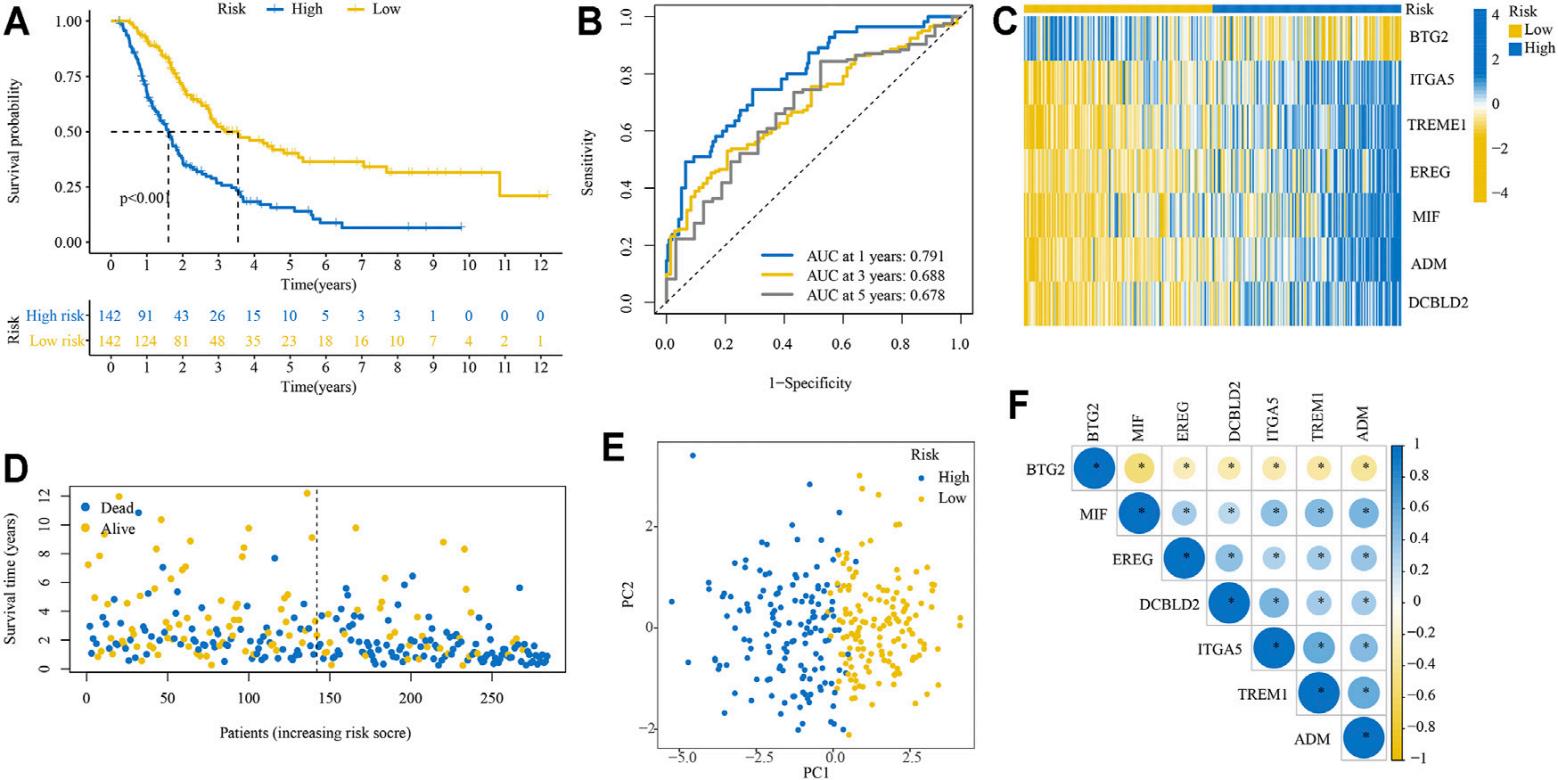
Performance of the IRRGs prognostic signature in training cohort. **(A)** Kaplan–Meier curve shows OS differences between the high- and low-risk groups. **(B)** ROC curve for 1, 3, and 5-years OS. **(C)** Expression levels of seven signature genes in high- and low-risk groups. **(D)** The distribution of survival status. **(E)** PCA plot. **(F)** Correlation of seven signature genes.

The Kaplan-Meier survival analysis showed that patients in the high-risk group had significantly unfavorable OS compared with those in the low-risk group (Figure 3A). The predictive efficacy of the risk score for OS was further evaluated by ROC analysis, and the area under the ROC curve (AUC) was 0.791, 0.688, and 0.678 for 1, 3, and 5-years, respectively (Figure 3B). Expression levels of the seven signature genes were significantly different between high- and low-risk groups (Figure 3C). Patients with a high-risk score had a higher death probability than those with a low-risk score (Figure 3D). The high- and low-risk groups displayed different distributed patterns in a PCA plot (Figure 3E). Correlation analysis indicated that the seven signature genes were significantly correlated with each other (Figure 3F).

### Verification of the IRRGs Prognostic Signature in 10 PDAC Cohorts

**FIGURE 4:**
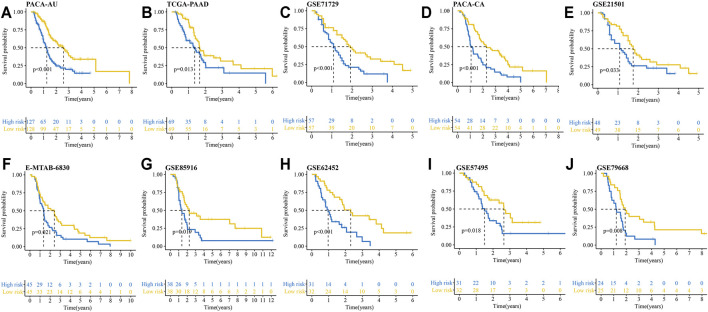
Kaplan–Meier curves show OS differences between the high- and low-risk groups in 10 PDAC cohorts. **(A)** PACA-AU cohort. **(B)** TCGA-PAAD cohort. **(C)** GSE71729 cohort. **(D)** PACA-CA cohort. **(E)** GSE21501 cohort. **(F)** E-MTAB-6830 cohort. **(G)** GSE85916 cohort. **(H)** GSE62452 cohort. **(I)** GSE57495 cohort. **(J)** GSE79668 cohort.

We then verified the reliability and capability of the IRRGs signature in 10 PDAC cohorts. Kaplan-Meier survival analysis showed the high-risk group had a significantly worse prognosis compared to the low-risk group in all 10 cohorts (Figures 4A–J).

**FIGURE 5:**
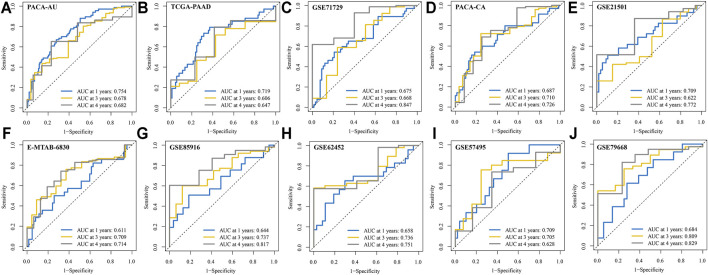
ROC curves for 1, 3, and 4-years OS in the 10 validation cohorts. **(A)** PACA-AU cohort. **(B)** TCGA-PAAD cohort. **(C)** GSE71729 cohort. **(D)** PACA-CA cohort. **(E)** GSE21501 cohort. **(F)** E-MTAB-6830 cohort. **(G)** GSE85916 cohort. **(H)** GSE62452 cohort. **(I)** GSE57495 cohort. **(J)** GSE79668 cohort.

We conducted ROC analysis for 1, 3, and 4-years OS in the 10 validation cohorts to further validate the reliability of the signature. The AUC for 1, 3, and 4-years OS in the 10 PDAC cohorts were greater than 0.600 (Figures 5A–J). Notably, the AUC of ROC curve for 4-years OS reached 0.847, 0.829, and 0.817 in GSE71729, GSE79668, and GSE85916 cohorts, respectively. The C-index of our signature was greater than 0.600 at different survival times except for in the TCGA-PAAD cohort (Supplementary Figure S2). Additionally, compared with six previously published gene signatures, our IRRGs signature had the highest C-index in 9 of 11 PDAC cohorts.

**FIGURE 6:**
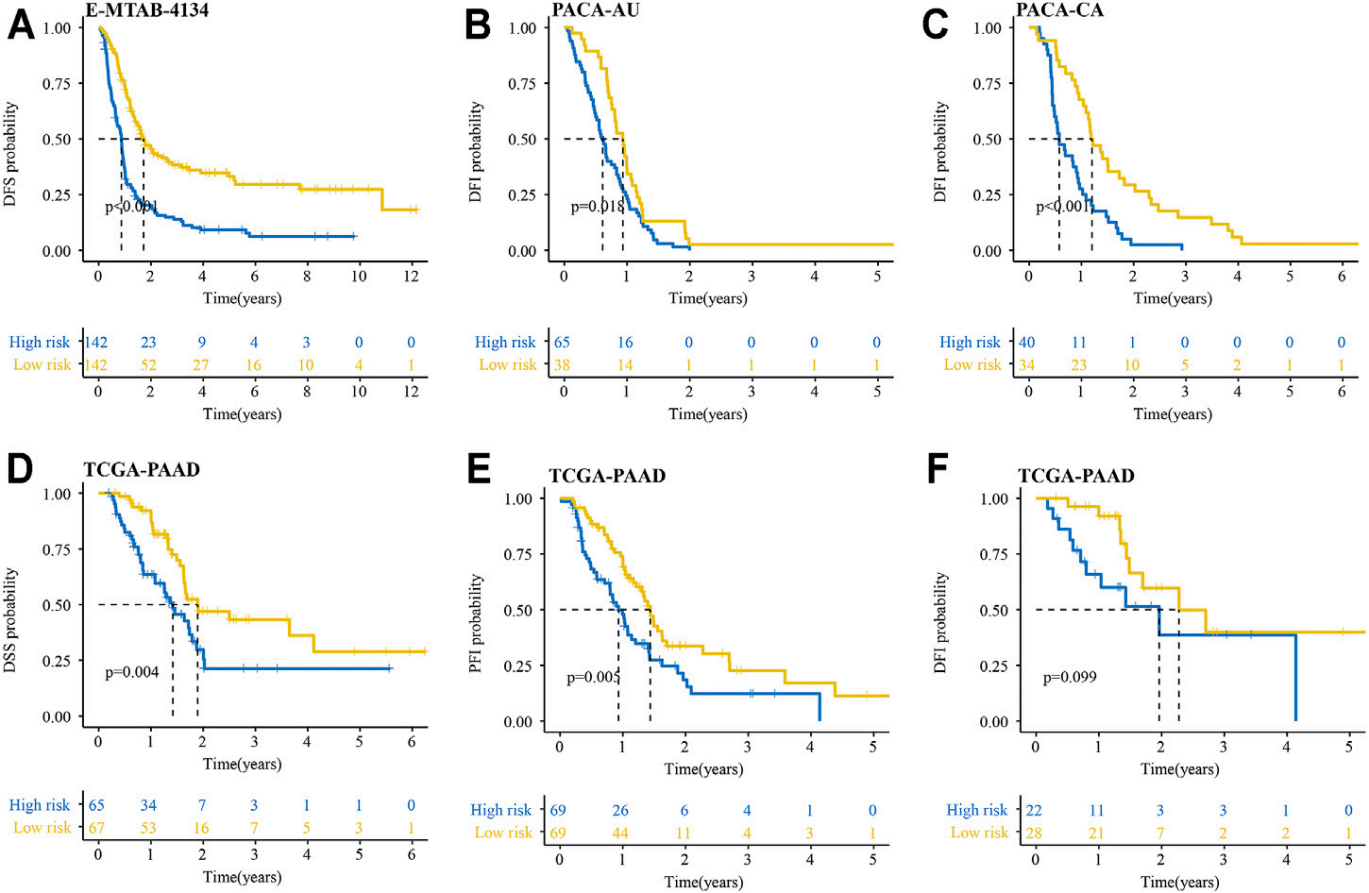
Performance of the IRRGs signature in the aspect of DFS, DFI, DSS, and PFI. **(A)** DFS difference in high- and low-risk patients in E-MTAB-6134 cohort. **(B,C)** DFI differences in high- and low-risk patients in PACA-AU **(B)** and PACA-CA **(C)** cohorts. **(D–F)** DSS **(D)**, PFI **(E)**, and DFI **(F)** differences in high- and low-risk patients in TCGA-PAAD cohort.

We also explored whether our signature could be used for prediction of other important survival outcomes, including disease-free survival (DFS), disease-free interval (DFI), disease-specific survival (DSS), and progression-free interval (PFI). Kaplan-Meier survival analysis showed the patients with high-risk had a significantly worse DFS than their low-risk counterparts in the E-MTAB-6134 cohort (Figure 6A). In the PACA-AU and PACA-CA cohorts, patients with high-risk had a significantly worse DFI than those with low-risk (Figures 6B,C). In the TCGA-PAAD cohort, patients with high-risk had a significantly worse DFI and DSS than those with low-risk (Figures 6D,E). However, there were no significant differences in DFI between two groups in TCGA-PAAD cohort (Figure 6F).

### Prognostic Value of the IRRGs Signature Was Independent of Resection Margin, Tumor Stage, and Grade

**FIGURE 7:**
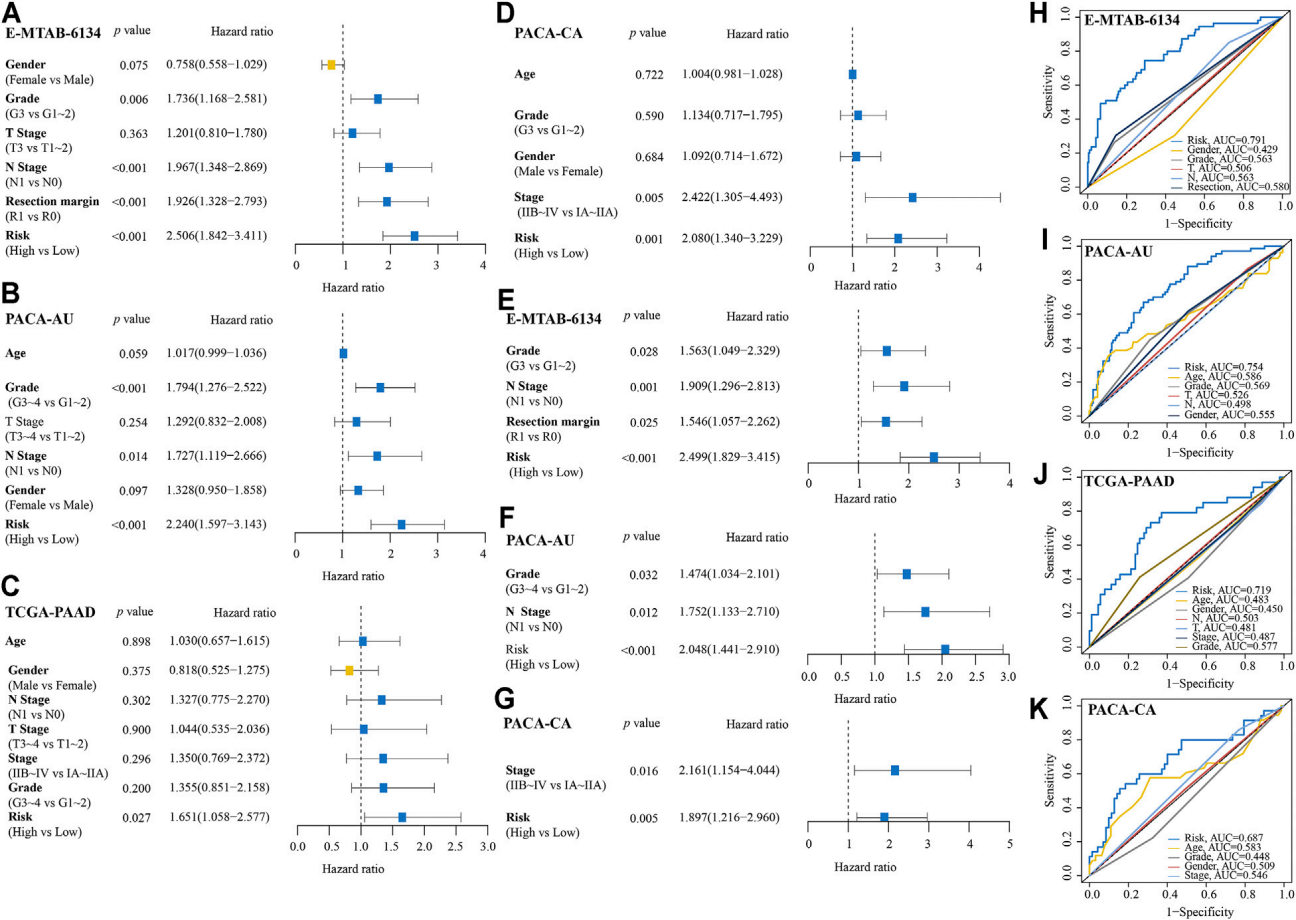
Comparison of IRRGs prognostic signature and other clinical variables. **(A–D)** Univariate Cox regression analyses of the IRRGs signature and other clinical variables. **(E–G)** Multivariate Cox regression analyses of the IRRGs signature. **(H–K)** Compared the predictive accuracy of risk score and other clinical characteristics for 1-year OS by ROC curve.

In the univariate Cox analyses, high-risk was significantly correlated with poor OS (E-MTAB-6134 cohort: HR = 2.506, 95% CI = 1.842–3.411, *p* < 0.001; PACA-AU cohort: HR = 2.240, 95% CI = 1.597–3.143, *p* < 0.001; TCGA-PAAD cohort: HR = 1.651, 95% CI = 1.058–2.577, *p* = 0.027; PACA-CA cohort: HR = 2.080, 95% CI = 1.340–3.229, *p* = 0.001) (Figures 7A–D). The multivariate Cox analyses indicated that high-risk was a hazardous factor for OS after correcting for other significant clinical factors (E-MTAB-6134 cohort: HR = 2.499, 95% CI = 1.829–3.415, *p* < 0.001; PACA-AU cohort: HR = 2.048, 95% CI = 1.441–2.910, *p* < 0.001; PACA-CA cohort: HR = 1.897, 95% CI = 1.261–2.960, *p* = 0.005) (Figures 7E–G). ROC analyses showed that the signature had good predictive accuracy in OS of PDAC patients than other clinical variables (Figures 7H–K).

**FIGURE 8:**
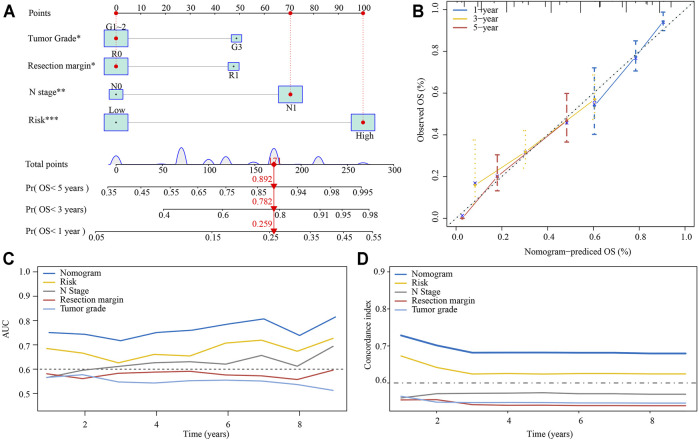
Establishment of a nomogram predicting the OS for PDAC patients. **(A)** The nomogram was built based on tumor grade, resection margin, N stage, and risk. **(B)** Calibration curves of the nomogram. **(C)** Time-varying AUC of the nomogram and four single factors. **(D)** Time-varying C-index of the nomogram and four single factors.

Based on the multivariate analysis results of E-MTAB-6134 cohort, we established a nomogram which could further improve survival predictive ability for PDAC patients. A total of four factors were integrated in the nomogram to predict the OS of PDAC patients (Figure 8A). The total points were calculated by adding up the corresponding points of each factor. The calibration curve suggested that the nomogram that predicted the survival rate was close to the actual situation for the 1-, 3-, and 5-year survival (Figure 8B). The time-varying AUC and C-index plots indicated that the predictive ability of the nomogram was better than that of any single factor (Figures 8C,D).

### Gemcitabine Response and Risk Score

**FIGURE 9:**
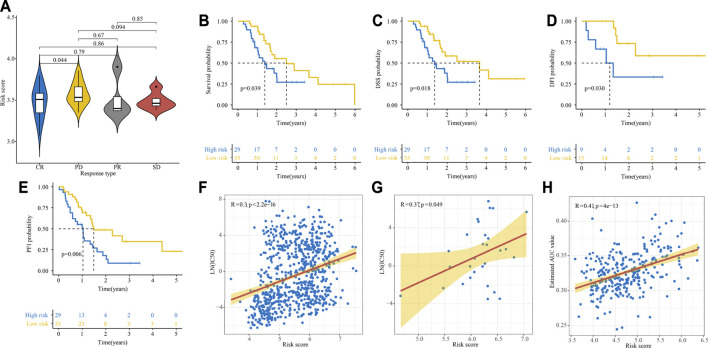
Relationship between gemcitabine response and risk score. **(A)** Relationship between risk score and response to gemcitabine in TCGA cohort. **(B–E)** Kaplan–Meier curve of OS **(B)**, DSS **(C)**, DFI **(D)**, and PFI **(E)** for high- and low-risk patients treated with gemcitabine. **(F, G)** Relationship between cells’ risk score and gemcitabine IC50 in 808 cancer cells **(F)** and 29 PDAC cells **(G)** in GDSC database. **(H)** Relationship between risk score and estimated AUC value of gemcitabine calculated by the pRRophetic algorithm.

We conducted a subgroup analysis of 62 patients treated with gemcitabine in TCGA-PAAD cohort. As shown in Figure 9A, patients presented a CR had significantly lower risk score compared with those presented a PD. The survival analyses showed that patients in the high-risk group had significantly poor OS, DSS, DFI, and PFI as compared with those in the low-risk group (Figures 9B–E). By analyzing gene expression and drug sensitivity data in the GDSC database, we revealed a significant positive association between cancer cells’ risk score and gemcitabine IC50, indicating that patients with higher risk score were more likely to be resistant to gemcitabine (Figures 9F,G). We unutilized the R package pRRophetic, which had a built-in ridge regression model, to predict the drug response of patients in E-MTAB-6134 cohort based on their gene expression profiles. The estimated AUC value of each compound in each sample was obtained. As a result, a total of 354 compounds and their estimated AUC value of each patient in E-MTAB-6134 cohort were yielded and listed in Supplementary Table S7. We found that there was significantly positive correlation between risk score and AUC value of gemcitabine (Figure 9H).

### Identification of Potential Drugs for Patients with High-Risk Score

**FIGURE 10:**
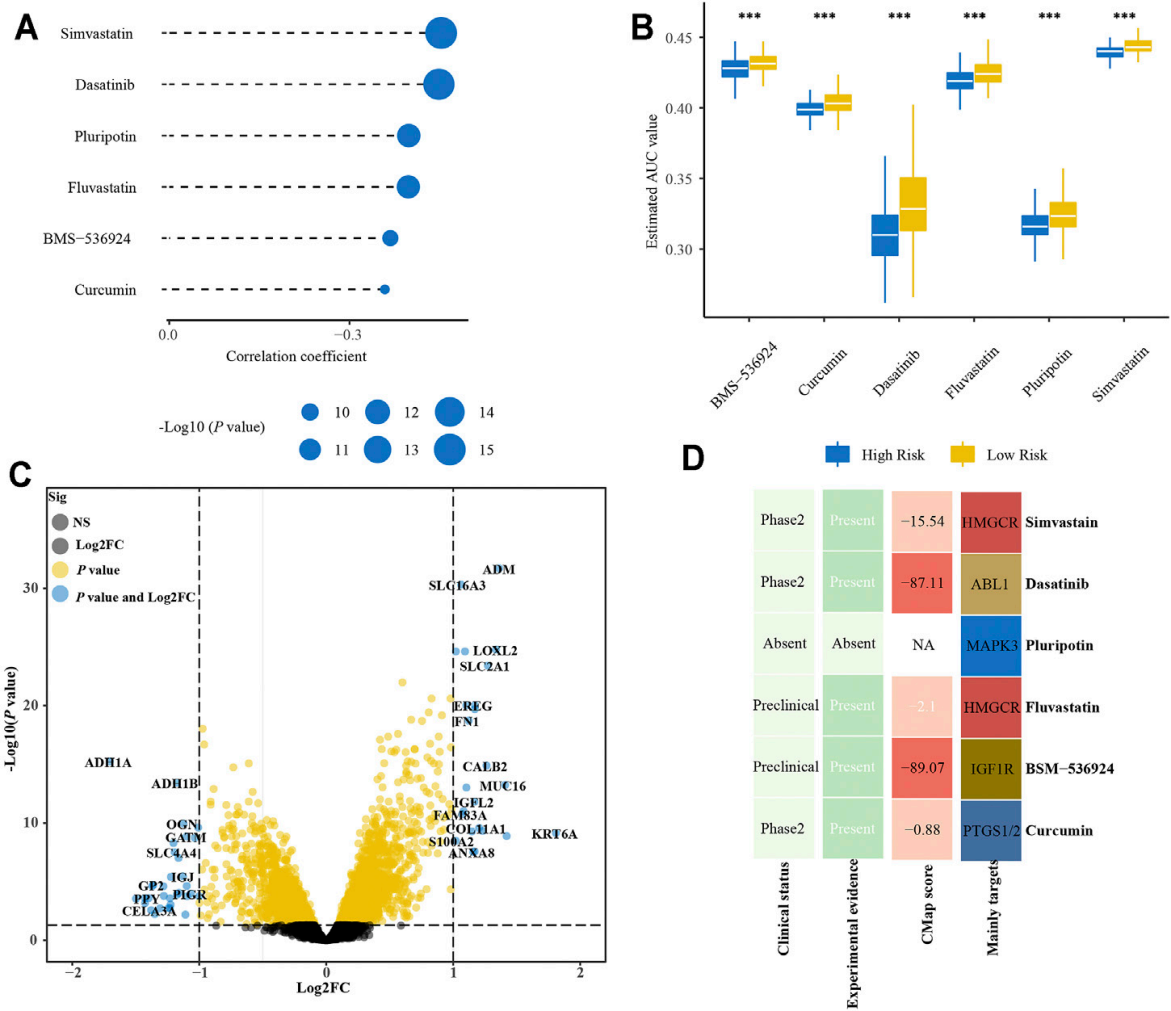
Identification of promising therapeutic drugs for high-risk patients. **(A)** Correlation analysis of estimated AUC value of six compounds and risk score. **(B)** Comparison of estimated AUC value of six compounds between high- and low-risk groups. **(C)** Volcano plot of differential expression genes between high- and low-risk groups. **(D)** Clinical and experimental evidence of six compounds in the treatment of PDAC and their CMap scores.

We further analyze the results in Supplementary Table S6 to identify potential drugs for patients with a high-risk score. Correlation analysis between AUC value and risk score was conducted to pick compounds with negative correlation coefficient (R < −0.3). Differential analysis was then carried out between high- and low-risk groups to select compounds with lower AUC values in the high-risk group (*p* < 0.001 and mean_high_/mean_low_ < 0.98). Collectively, these two analyses yielded six compounds (simvastatin, dasatinib, pluripotin, fluvastatin, BMS-536924, curcumin) (Figures 10A,B). Furthermore, there were 23 up-regulated and 34 down-regulated genes in the high-risk group as compared with the low-risk group in E-MTAB-6134 cohort (Figure 10C). The CMap analysis was then used to identify compounds of which gene expression patterns were oppositional to the expression patterns of the high-risk group (i.e., gene expression increased in the high-risk group tend to be decreased by the perturbagen of certain compounds). We found that BMS-536924 and dasatinib had CMap scores <−87 (Figure 10D), indicating that these two drugs had great potential for the treatment of high-risk patients.

### Frequencies of TP53, KRAS, and CDKN2A Mutations in High- and Low-Risk Groups

**FIGURE 11:**
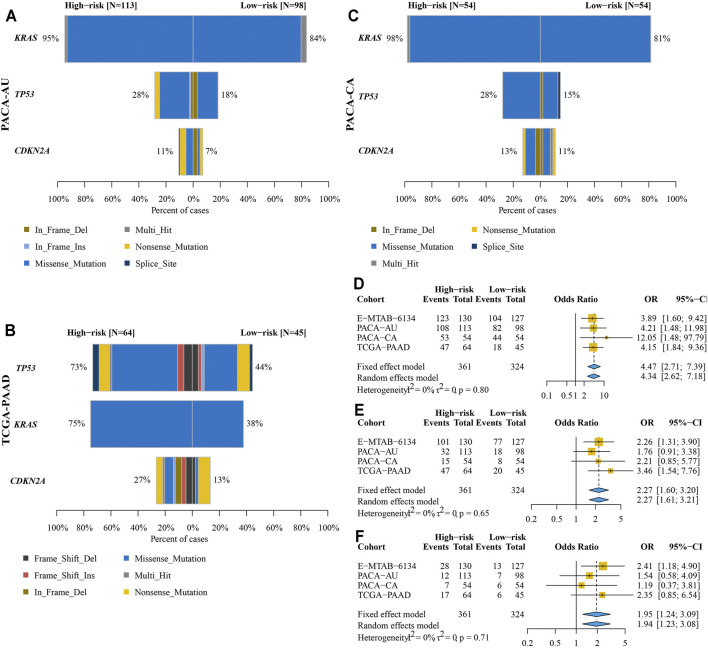
Comparison of KRAS, TP53, and CDKN2A mutations between high- and low-risk groups. **(A–C)** Frequencies and types of KRAS, TP53, and CDKN2A mutations in PACA-AU **(A)**, PACA-CA **(B)**, and TCGA-PAAD **(C)** cohorts. **(D–F)** Pooled analysis to compare the overall frequencies of KRAS **(D)**, TP53 **(E)**, and CDKN2A **(F)** mutations between high- and low-risk groups.

We found that KRAS mutation had the most frequency in PACA-AU, PACA-CA, and E-MTAB-6134 cohorts, while TP53 mutation was the most common in TCGA-PAAD cohort (Figures 11A–C). The frequency of KRAS mutation were significantly higher in the high-risk group in all four cohorts, while the frequency of TP53 mutation was significantly higher in the high-risk group in E-MTAB-6134 and TCGA-PAAD cohorts. However, the frequency of CDKN2A mutation was significantly higher in the high-risk group only in E-MTAB-6134 cohort. We next conducted a pooled analysis to compare the overall frequencies of TP53, KRAS, and CDKN2A mutations between the high- and low-risk groups in four cohorts. We found that frequencies of TP53, KRAS, and CDKN2A mutations were significantly higher in the high-risk group compared with the low-risk group (KRAS: OR = 4.47, 95% CI = 2.71–7.39, *p* < 0.001; TP53: OR = 2.27, 95% CI = 1.60–3.20, *p* < 0.001; CDKN2A: OR = 1.95, 95% CI = 1.24–3.09, *p* < 0.001) (Figures 11D–F).

### Immune Infiltration Estimations and GSEA Analyses

**FIGURE 12:**
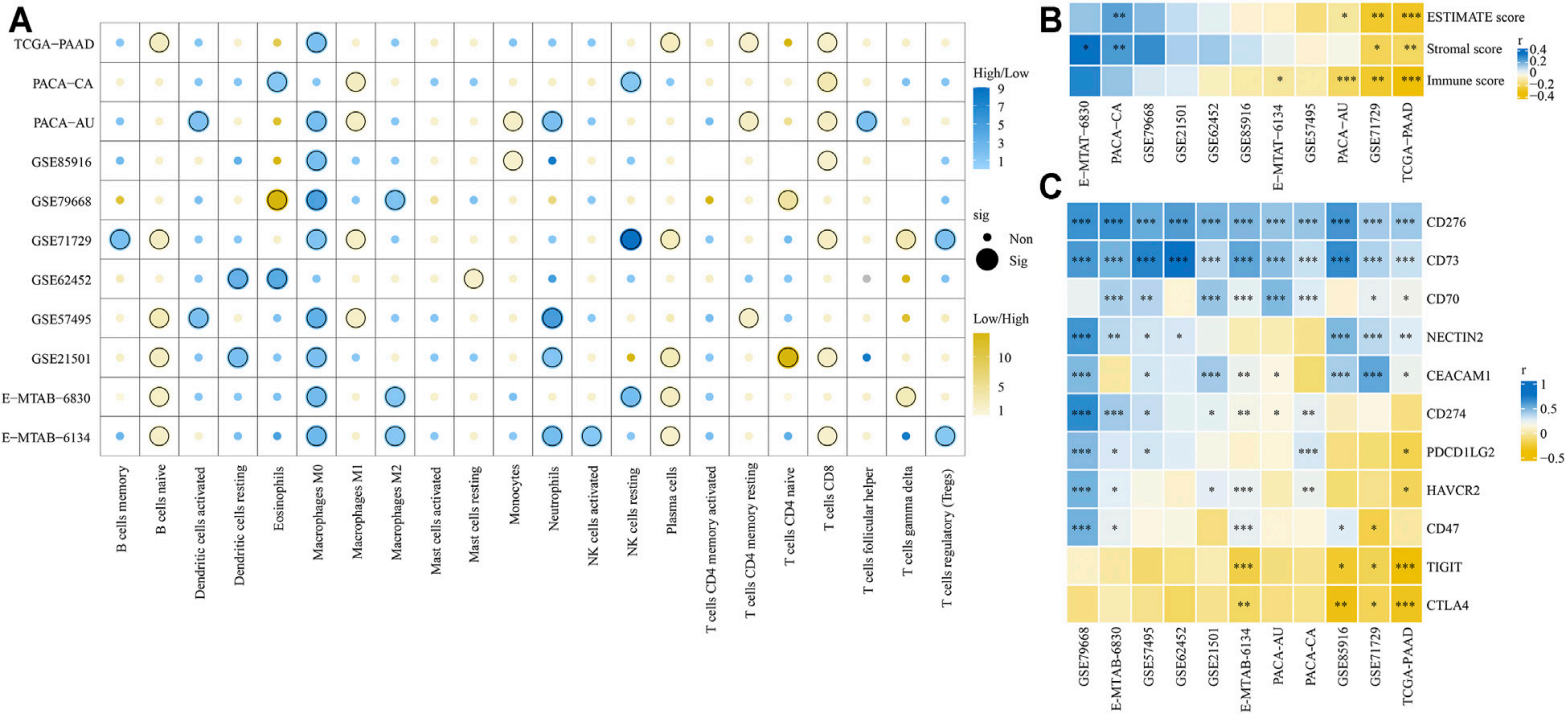
Comparison of tumor immune microenvironment between high- and low-risk groups. **(A)** Infiltration of 22 types of immune cells between high- and low-risk groups was compared. **(B)** Correlation of risk scores and ESTIMATE, stromal, and immune scores. **(C)** Correlation of risk scores and expression levels of 11 immune checkpoints. *p < 0.05, **p < 0.01, ***p < 0.001.

Based on the CIBERSORT algorithm, we compared the proportion of 22 types of immune cells between high- and low-risk groups. As shown in Figure 12A, CD8^+^ T, naïve B, plasma, and macrophages M1 cells were significantly higher in the low-risk groups, while macrophages M0, neutrophils, and macrophages M2 cells were more likely higher in the high-risk groups. Next, we used ESTIMATE algorithm to calculate stromal and immune scores of each tumor sample. We found that the immune scores were significantly negative correlation with risk scores in TCGA-PAAD, E-MTAB-6134, GSE71729, and PACA-AU cohorts, while the stromal scores were significantly positive correlation with risk scores in E-MTAB-6134 and PACA-CA cohorts (Figure 12B). We also explored the correlation of risk scores and expression of 11 well-documented immune checkpoints. We found that expression levels of immune checkpoints were significantly positive correlation with risk scores in most cohorts except for TIGIT and CTLA4 (Figure 12C). Notably, CD73 and CD276 were extremely positive correlation with risk scores in all 11 cohorts. GSEA analysis indicated that most of the 50 hallmark gene sets were upregulated in high-risk groups although there were some differences among the 11 cohorts (Supplementary Figure S3). Especially, the glycolysis and hypoxia gene sets were significantly upregulated in the high-risk groups compared with low-risk groups in 10 cohorts.

## Discussion

With the development of high throughput sequencing technology, there has been an increasing interest in constructing gene signatures to predict the prognosis of patients with PDAC and other malignant tumors. Previously, several valuable PDAC prognostic signatures have been constructed by using bioinformatics analysis. For example, signatures based on glycolysis, m6A, ferroptosis, immune, and extracellular vesicle predict 1-year OS for PDAC with AUC at 0.805, 0.736, 0.81, 0.755, and 0.640, respectively, which were similar to our study (Xu et al., 2020; Chen et al., 2021; Song et al., 2021; Xu et al., 2021; Yuan et al., 2021). However, the number of patients included in the abovementioned studies ranged from 183 to 441 and were relatively low. As described in their publications, the applicability of these signatures warrants further validation in larger PDAC cohorts. In the present work, we developed and validated an IRRGs signature for prognostic risk stratification and prediction based on 11 PDAC cohorts including 1337 PDAC patients. The prognosis of the high-risk group was significantly worse than its low-risk group counterpart. The AUC for OS was greater than 0.600 in all 11 PDAC cohorts, highlighting the good performance and accuracy of our signature. Furthermore, compared with six previous published gene signatures, our IRRGs signature had the highest C-index in 9 of 11 PDAC cohorts.

Our signature consisted of seven IRRGs, including ITGA5, TREM1, EREG, ADM, MIF, DCBLD2, and BTG2. It has been reported that ITGA5 overexpression was inversely correlated with OS of PDAC patients, and inhibition of ITGA5 potentiated the cytotoxicity of gemcitabine (Kuninty et al., 2019). Preclinical study showed that blockade of TREM1 specifically suppresses key cytokines and thereby inhibited tumor growth in human pancreatic cancer xenografts and prolonged the survival of mice (Shen and Sigalov, 2017). An experimental study has suggested that EREG contributed to the progression of pancreatic cancer (Zhu et al., 2000). The serum level of adrenomedullin, a peptide hormone encoded by the ADM gene, was significantly increased in patients with PDAC compared to chronic pancreatitis and healthy individuals (Keleg et al., 2007). Knockdown of ADM in pancreatic tumor-bearing mice significantly inhibited the recruitment of myelomonocytic cells and tumor angiogenesis (Xu et al., 2016). Multiple studies have documented that elevated MIF expression was associated with increased tumor aggressiveness, reduced sensitivity to gemcitabine, and worse survival in PDAC patients (Wen et al., 2021). Additionally, a previous study has identified DCBLD2 as a prognostic and diagnostic biomarker in PDAC (Feng et al., 2021b). However, the AUC values of DCBLD2 for 1-year OS prediction in their publication were 0.708, 0.753, and 0.690 in MTAB-6134, PACA-AU, and TCGA-PAAD cohorts, respectively, which were lower than the values in our study. Besides, the AUC values of DCBLD2 for OS prediction in other PDAC cohorts have not been reported in their publication, indicating that the predictive performance of DCBLD2 as a single-gene prognostic indictor needs to be further verified. Furthermore, in line with previous studies that reported BTG2 as a tumor suppressor involved in multiple biological processes of cancer (Mao et al., 2015), our study identified BTG2 as a prognostic protection gene in PDAC. Existing literature have also revealed that miR-27a promoted PDAC cell growth and migration via directly targeted BTG2 (Frampton et al., 2014; Shang et al., 2020).

Gemcitabine has been the cornerstone of PDAC treatment in all stages and gemcitabine resistance is currently the main problem of chemotherapy for PDAC patients. Our study found that high-risk PDAC patients had significantly low response rate to gemcitabine than low-risk patients, indicating that our signature may be used for predicting gemcitabine response of PDAC patients. We identified two agents that might have potential therapeutic implications for high-risk patients who might be resistant to gemcitabine. BMS-536924, a small molecule inhibitor of the IGF-1 receptor, has been confirmed in preclinical study to have a broad spectrum of antitumor activity *in vitro* and *in vivo* (Huang et al., 2009)*.* Studies also have revealed that IGF1R contributed to tumor growth and gemcitabine resistance in PDAC (Tian et al., 2013; Subramani et al., 2014). Clinical trials showed that anti-IGRF1R antibody combination with gemcitabine was associated with improvement in OS as compared with gemcitabine combination with erlotinib, indicating that anti-IGRF1R might be a promising treatment strategy for PDAC patients (Abdel-Wahab et al., 2018). Preclinical studies have documented that dasatinib inhibits tumor growth and metastasis in mouse models of PDAC (Morton et al., 2010; Nagaraj et al., 2010). Unfortunately, dasatinib as monotherapy or combination therapy have failed to demonstrate clinical benefit in patients with PDAC (Garcia-Sampedro et al., 2021). Nevertheless, clinical trials evaluating the efficacy of dasatinib for PDAC treatment are still ongoing (NCT01652976 and NCT02465060). Our work might provide new insights into improving therapeutic effect of dasatinib by selecting potential dasatinib-responsive patients.

Furthermore, we aimed to explain the reason of differences in prognosis between high- and low-risk groups from the views of gene mutation and immune cell infiltration. It has been generally accepted that KRAS, TP53, and CDKN2A were three of the most frequently mutated genes in PDAC patients (Cicenas et al., 2017). A study has reported that the OS of PDAC patients without TP53 and KRAS mutations was more than twice as long as that of patients with TP53 and KRAS mutations (Masetti et al., 2018). Additionally, PDAC patients without TP53 mutation were disease-free for 1.51 times longer than those with TP53 mutation (Li et al., 2019). Patients without CDKN2A mutation had significantly increased OS compared to those with CDKN2A mutations, indicating that CDKN2A mutation was an independent negative prognostic OS indicator for PDAC patients. We found that the frequencies of KRAS, TP53, and CDKN2A mutations in high-risk patients with poor prognosis were significantly higher than that in low-risk patients, which were in accord with previous studies.

Inflammation induces an immunosuppressive tumor microenvironment and thereby drives tumor growth, progression, and metastasis (Greten and Grivennikov, 2019). We revealed that the high-risk group had increased infiltration of macrophages M0, neutrophils, and macrophages M2 cells, while the low-risk group had increased infiltration of CD8^+^ T, naïve B, plasma, and macrophages M1 cells. A study has reported that CD8^+^ T cells infiltration was associated with longer survival in PDAC patients (Hou et al., 2019). Similarly, a high number of CD8^+^ lymphocytes in tumor samples was significantly associated with longer DFS and OS of PDAC patients (Lohneis et al., 2017). Neutrophil cells, the most abundant leukocytes in the circulation, play an important role in inflammation and immune responses (Rosales, 2018). CD8^+^ T lymphocytes could be suppressed by tumor-induced neutrophil cells, and inhibition of neutrophils accumulation by lorlatinib attenuates PDAC growth (Coffelt et al., 2015; Nielsen et al., 2021). Evidence came from previous studies showed that higher intratumoral B cells density was associated with good prognosis in multiple cancers including PDAC (Fridman et al., 2020). Additionally, we found the expressions of two immune checkpoints, CD276 and CD73, were significantly positively associated with risk scores in all 11 PDAC cohorts, while expressions of TIGIT and CTLA4 were negatively related to risk scores. CD276 has been reported to promote tumor progression by inhibiting the functions of NK and CD8^+^ T cells, and its expression levels were associated with poor prognosis in multiple cancers (Zang et al., 2007; Crispen et al., 2008; Zhang et al., 2009; Lee et al., 2017; Inamura et al., 2018). Meanwhile, a recent preclinical study has found that targeting CD276 by CAR-T cells effectively inhibited PDAC tumor growth *in vitro* and *in vivo* (Du et al., 2019), suggesting the potential efficacy in a selected subgroup of PDAC patients. Furthermore, CD73 has emerged as an attractive therapeutic target for immunotherapy and has been reported to promote gemcitabine resistance in PDAC cells (Harvey et al., 2020; Yu X. et al., 2021).

The GSEA analysis indicated that hypoxia and glycolysis pathways were upregulated in the high-risk group. Recent studies have documented that hypoxia and glycolysis-related gene signatures were associated with tumor microenvironment and might be used to predict the prognosis of PDAC patients (Ding et al., 2021; Song et al., 2021). Hypoxia has been considered as an indicator of the inflamed tumor microenvironment and leads to activation of tumor-promoting inflammatory responses (Triner and Shah, 2016). The associations among inflammation, hypoxia, and glycolysis have been well documented in previous studies. On one hand, inflammatory cells tend to switch their metabolism toward glycolysis to meet their high energetic demand (Soto-Heredero et al., 2020); on the other hand, hypoxia inducible factor-1α induces an increased expression of glycolytic enzymes, which contributes to maintaining bioenergetic homeostasis during hypoxia (Kierans and Taylor, 2021).

We recognized several limitations in this study. First, we only selected IRRGs from two gene sets, which might neglect other important prognostic IRRGs not included in these two sets. Second, the correlation of gemcitabine response and our signature should be further validated in more PDAC patients. Third, all the conclusions in this study were drawn from *in silico* analyses, and further experimental or clinical validations to increase the evidence level of our findings were needed. Thus, we will design multicenter prospective clinical trials with large sample sizes for further verification in future work.

## Conclusion

In conclusion, we developed and verified a signature containing seven IRRGs to predict the survival outcome of patients with PDAC. Further analysis indicated that the signature could be used to predict responses of PDAC patients to gemcitabine treatment. We also combined the IRRGs signature with traditional clinicopathological features to construct a nomogram with more accurate survival predictive ability. For patients with high-risk scores, our study provided them with potential therapeutic drugs, which might effectively improve their prognosis. Nevertheless, further prospective studies on the large, well-performed PDAC cohorts are needed to validate the stability of our IRRGs signature and to increase its evidence level.

## Data Availability

Publicly available datasets were analyzed in this study. This data can be found here: TCGA-PAAD (*n* = 138) cohort was obtained from UCSC Xena (https://xenabrowser.net/datapages/). PACA-AU (*n* = 255) and PACA-CA (*n* = 108) cohorts were obtained from ICGC database (https://dcc.icgc.org/). GSE71729 (*n* = 114), GSE21501 (*n* = 97), GSE85916 (*n* = 76), GSE57495 (*n* = 63), GSE62452 (*n* = 63), GSE79668 (*n* = 49), GSE15471 (*n* = 72), GSE16515 (*n* = 32), and GSE172356 (*n* = 62) cohorts were obtained from GEO database (https://www.ncbi.nlm.nih.gov/geo/). E-TMAB-6134 (*n* = 284) and E-MTAB-6830 (*n* = 90) cohorts were obtained from ArrayExpress database (https://www.ebi.ac.uk/arrayexpress).
