# Supplementary material for: An Inflammatory Response Related Gene Signature Associated with Survival Outcome and Gemcitabine Response in Patients with Pancreatic Ductal Adenocarcinoma
Source: Front Pharmacol. 2021 Dec 23;12:778294. doi: 10.3389/fphar.2021.778294 (PMC8733666; doi:10.3389/fphar.2021.778294)
Supplement: Supplementary file 1 [file DataSheet2.docx]

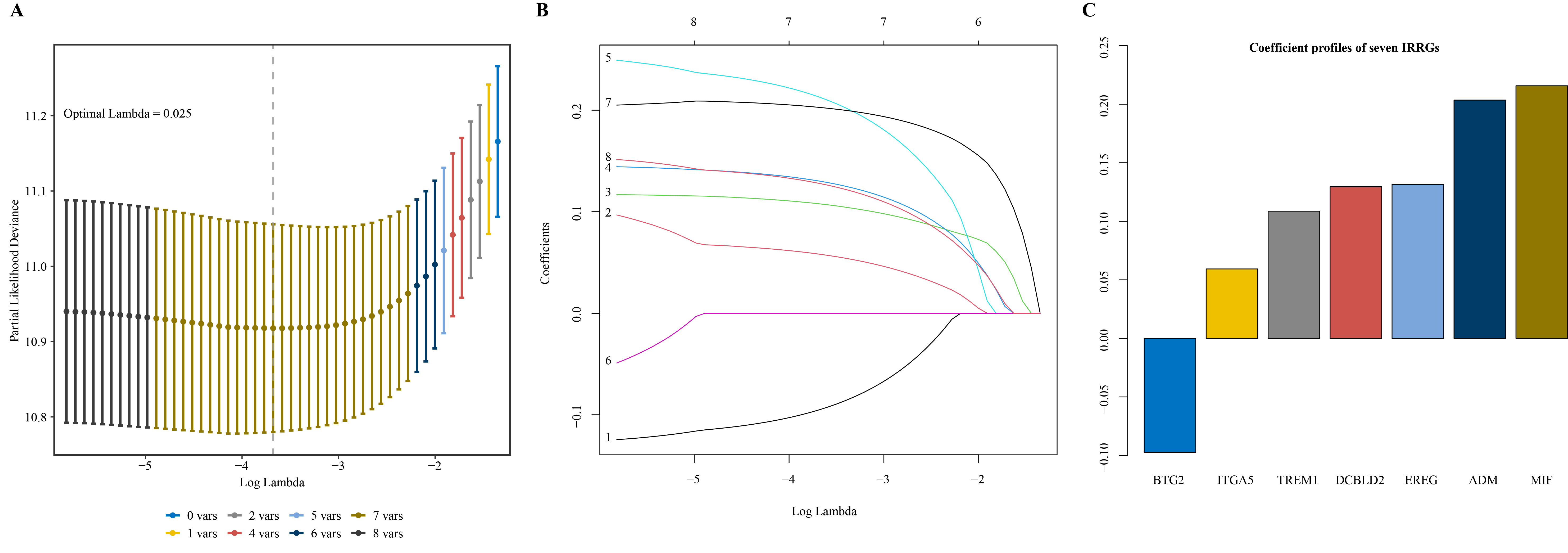


**FIGURE S1** Establishment of an IRRGs prognostic signature. (**A**) Cross-validated partial likelihood deviance. (**B**) Coefficient profiles of eight IRRGs at varying levels of λ. (**C**) Bar plot show coefficient profile of each gene.


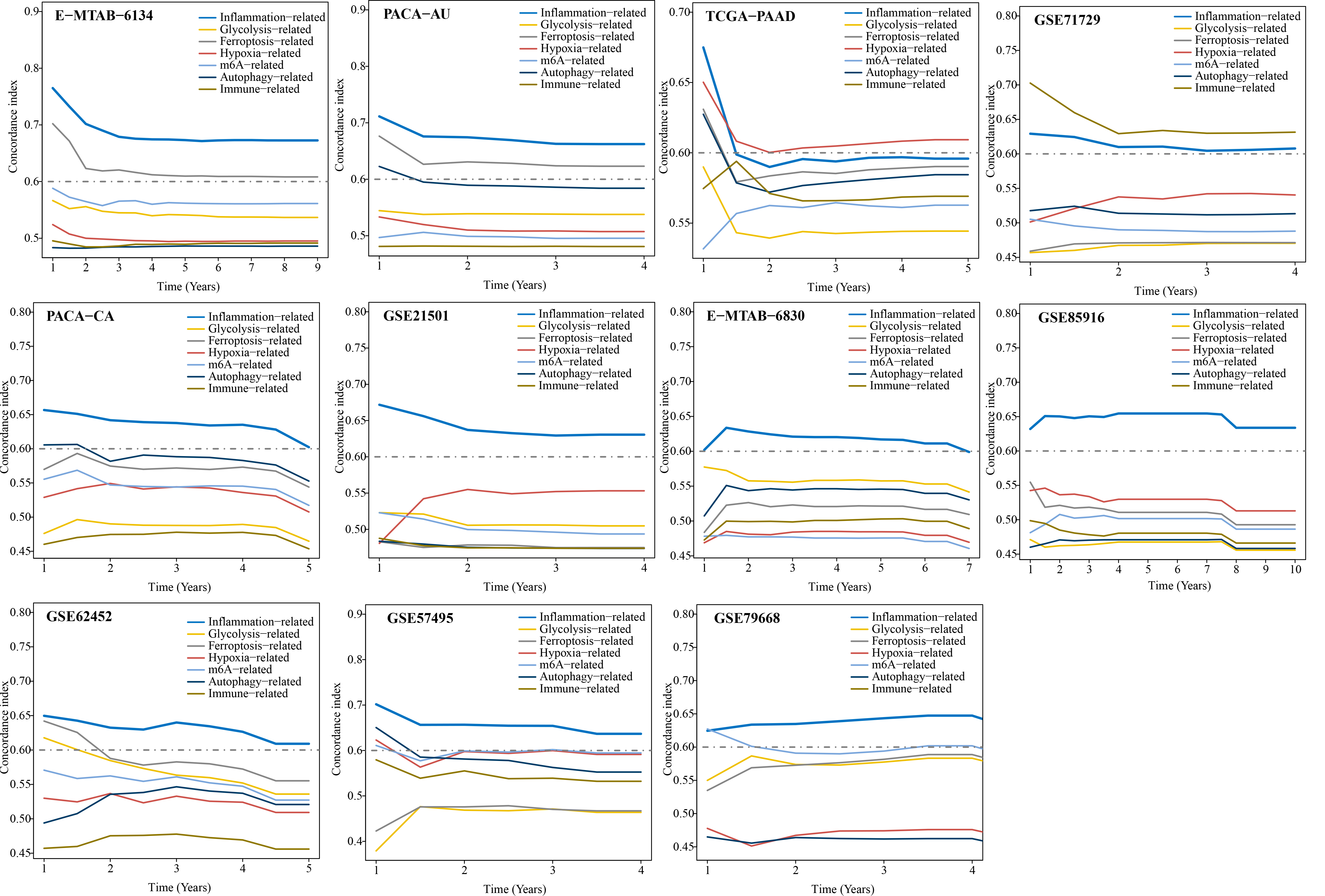


**FIGURE S2** Time-varying concordance index of our IRRGs signature and six previous published signatures.


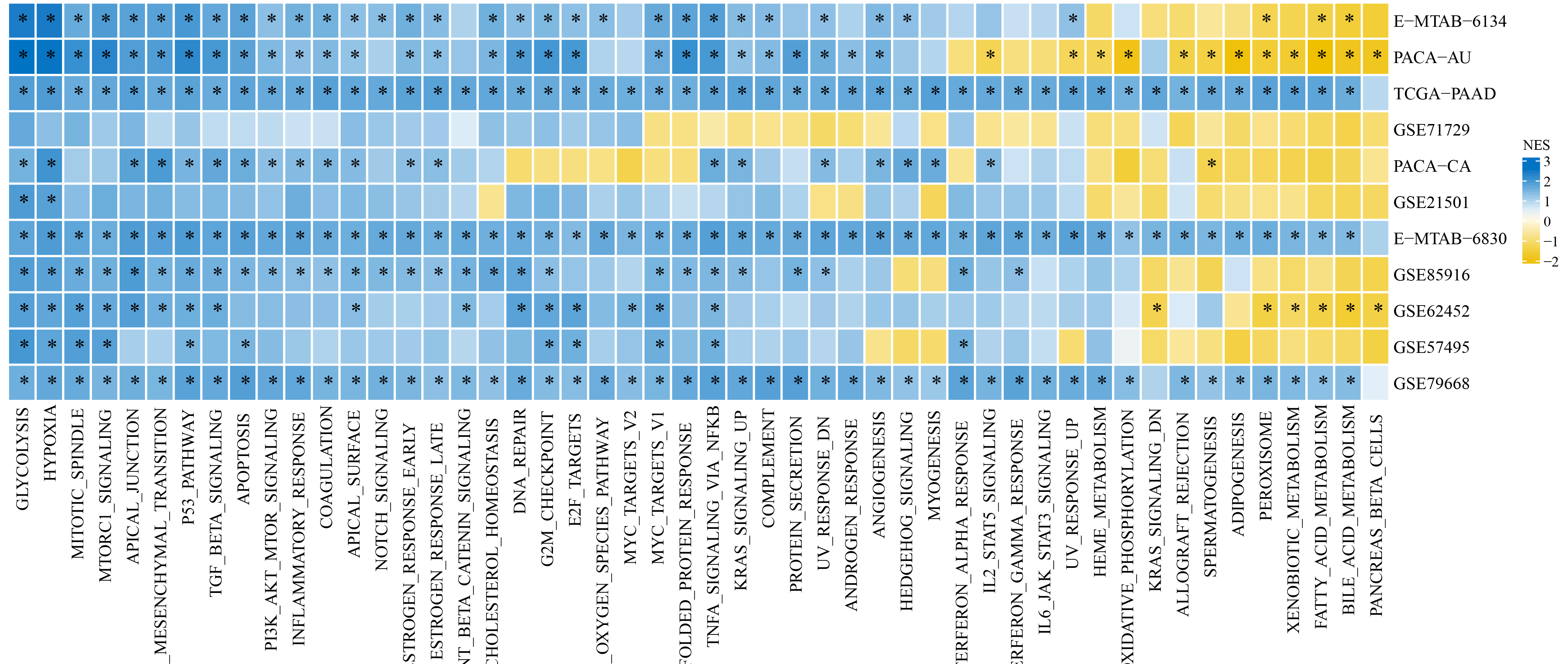


**FIGURE S3** The differences in 50 hallmark gene sets between high- and low-risk groups were compared by GSEA analysis. Blue represents upregulated in high-risk group, while yellow represents downregulated in high-risk group. * FDR<0.25
